# Supplementary figures and images for: Fusarium oxysporum infection-induced formation of agarwood (FOIFA): A rapid and efficient method for inducing the production of high quality agarwood
Source: PLoS One. 2022 Nov 4;17(11):e0277136. doi: 10.1371/journal.pone.0277136 (PMC9635754; doi:10.1371/journal.pone.0277136)

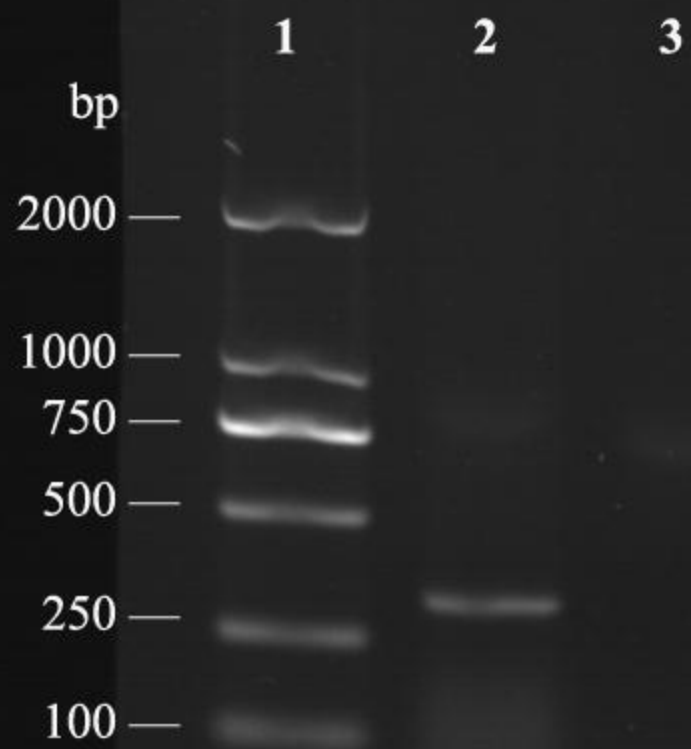

Supplement: S1 Raw images — (PDF) [file pone.0277136.s001.pdf]
