# Supplementary material for: Fusarium oxysporum infection-induced formation of agarwood (FOIFA): A rapid and efficient method for inducing the production of high quality agarwood
Source: PLoS One. 2022 Nov 4;17(11):e0277136. doi: 10.1371/journal.pone.0277136 (PMC9635754; doi:10.1371/journal.pone.0277136)
Supplement: Attachment — Submitted filename: Response to reviewers.docx [file pone.0277136.s002.docx]

Response to reviewer #1: Dear reviewer, we appreciate for your valuable suggestions on the problems existing in the article. After reviewing your comments carefully, the following are my responses to your suggestions：

1. The title: did reports *Fusarium* *oxysporum* can induce the formation of agarwood, and we study on the basis of justifying the formation of the new technology to rapid and efficient high-quality aloes, and as a result, we are also in the title changed, more prominent we validate the technology of rapid and efficient, in order to more relevant to our research.
2. Line 309-318: We added this part in the discussion about how the isolate reported in this study are protecting wild *Aquilaria* trees，this was indeed not shown in the previous content, thank you for your advice.
3. Line 49-59: We mention that many kinds of extracts have been obtained from natural agarwood, and the toxicity and induced of these extracts to agarwood have been reported，and *Fusarium oxysporum* is highly virulent to *Aquilaria* was known, so that we chose this particular isolate for our study
4. Line 78: We have corrected the mistake, thanks for your suggestion.
5. Line 79: In the research process, we focused on the influence of *Fusarium oxysporum* on the quality of agarwood, and did not discuss other strains in depth. We think this is not the main research direction of this paper, which is also our follow-up research target.
6. Line 88-99: According to your advice, we have added the specific description of sample handling and improved the relevant information.
7. Line 88-99:As above, we have added relevant content.
8. Line 105-108: Amplified gene information has been supplemented.
9. Line 109: We have clarified the subsequent verification process in the article.
10. Line 122: Your comments are very detailed, and we have revised them. Thank you.
11. Line 128-140: We have supplemented the specific operation steps, which is very important for the integrity of our research.
12. At line 160, we have added corresponding contents. Thank you for your suggestions.
13. Figure 2: According to your suggestion, we have modified the picture in order to fully reflect the relevant information.
14. Line 264: The discussion part of the article is really limited, and we also hope that it can be helpful to relevant research without causing trouble, so we have added some justified and critically discussed as you suggested.
15. Line 286: As you mentioned earlier, the term "Novel" really does not capture the focus of our research. Therefore, we have reconsidered this aspect and thank you again for your preciseness
16. Line 297: We have corrected this statement to make it clearer and more valuable for reference. Thank you for your advice.

We appreciate again for your meticulous suggestions on this study, so that our paper has been better supplemented and modified. We look forward to your reply to our revised draft again, thank you! Best wishes!

Response to reviewer #2: Dear reviewer, Thank you very much for your affirmation and recognition of our research. We know that this technology may be of great value in production application, so we have made corresponding changes to your suggestions.

1. As for the title (line 1), in this paper, we focus on FOIFA this novel technology for the influence of the quality of aloes, also found that the technique can really improve the production of high quality aloes, so we think that the technology in the production of artificial aloes with innovative significance and application value, which is a kind of improvement of production methods of artificial agarwood.
2. At line 67, according to your suggestion, we have carried out detailed description and information provision for the isolated strains to ensure the reliability and credibility of the strains we used, which is very important for this technology. Your opinion also ensures the integrity of the method.
3. As for materials and methods, our description of some details is indeed insufficient, and we also refer to a lot of relevant literature published by the laboratory before, without considering a complete description of all materials and methods in this paper. In this revised draft, We have clearly explained the details of plants (line 86), genes (line 76), samples (line 88), HPLC (line 142) and extraction process (line 128) you mentioned, and hope the supplementary content can answer your doubts.
4. As for the results, as you mentioned, we have supplemented some missing key information data. Such as fungus in the process of separation of the screening data were added in the method (line 67), the data and results to organize and expounded, and the results of GC to complete interpretation (Table 1), indeed can explain the increase of the ratio W B is aloes quality enhancement, but also changed the image of legend, to complete our results, these have been revised in the manuscript, Thank you very much for asking these questions to ensure the integrity of the article.

We appreciate again for your meticulous suggestions on this study, so that our paper has been better supplemented and modified. We look forward to your reply to our revised draft again, thank you! Best wishes!

Response to reviewer #3: Dear reviewer, in this paper, we focus on FOIFA this novel technology for the influence of the quality of aloes, also found that the technique can really improve the production of high quality aloes, so we think that the technology in the production of artificial aloes with innovative significance and application value, which is a kind of improvement of production methods of artificial agarwood. Thanks a lot! Best wishes!
