# Supplementary material for: Fusarium oxysporum infection-induced formation of agarwood (FOIFA): A rapid and efficient method for inducing the production of high quality agarwood
Source: PLoS One. 2022 Nov 4;17(11):e0277136. doi: 10.1371/journal.pone.0277136 (PMC9635754; doi:10.1371/journal.pone.0277136)
Supplement: Attachment — Submitted filename: Response to reviewers.docx [file pone.0277136.s003.docx]

Response to reviewer #1: Dear reviewer, thank you for your careful review of our manuscript, and we are very sorry for some of the unintentional mistakes we caused in responding to your previous comments. We have carefully made the following changes to your comments:

1. Line 79-80: As you have said, there are many fungal isolates that we has isolated from surface-sterilized symptomatic *Aquilaria sinensis* trees. But unfortunately, after our preliminary screening, only the *F. oxysporum* isolate (AsFo20150101) showed significant effect, which is why our research revolves around this effective isolate,we also made modification in the article to eliminate the misunderstanding.
2. In Figure 2: We have inserted the accession number of the *F. oxysporum* isolate used in the study(MW880244.1) into the phylogenetic tree, we apologize for not noticing this in the previous revision.
3. Indeed, we did not thoroughly explore the potential mechanism of agarwood formation induced by *Fusarium* before. Therefore, we combined with the previous study to explore the possible mechanism of the *F. oxysporum* isolate for the results shown in the current investigation, hoping to supplement the missing content.

We appreciate again for your meticulous suggestions on this study, so that our paper has been better supplemented and modified. We look forward to your reply to our revised manuscript again, thank you! Best wishes!

Response to reviewer #2: Dear reviewer, thank you for your suggestion about the title modification, which makes our title more readable and easy to understand. Thank you again for your suggestion and wish you a happy life.

Response to reviewer #3: Dear reviewer, thank you for your affirmation of our research and work. We also hope that our research can be of some help to the application of agarwood. Thanks again and wish you happiness
